# Analysis of the clinicopathological characteristics of pulmonary sclerosing pneumocytoma (PSP)

**DOI:** 10.3389/fmed.2026.1850296

**Published:** 2026-07-15

**Authors:** Shijun Chen, Haitao Liu, Yanru Jiang, Hongfei Ci, Chengling Zhao

**Affiliations:** 1 Department of Pulmonary and Critical Care Medicine, The First Affiliated Hospital of Bengbu Medical University, Bengbu, Anhui, China; 2 Department of Pathology, The First Affiliated Hospital of Bengbu Medical University, Bengbu, Anhui, China

**Keywords:** clinicopathological features, computed tomography, immunohistochemistry, pulmonary sclerosing pneumocytoma (PSP), tumour of the lung

## Abstract

**Objective:**

This study aimed to review the clinical, imaging, pathological, and immunohistochemical features of pulmonary sclerosing pneumocytoma (PSP) and to highlight observations that may help distinguish it from other entities.

**Methods:**

This is a retrospective, single-center case series of 22 patients with pathologically confirmed cases of pulmonary sclerosing pneumocytoma (PSP) who underwent surgical resection from January 2022 to August 2025. Clinical records, chest computed tomography (CT) findings, histopathology, immunohistochemistry, surgical treatment, and follow-up data were reviewed descriptively.

**Result:**

The results showed that 19 of these 22 patients were female, and the average age was 54.4 years. Typical CT findings were well-defined solid or ground-glass nodules with progressive enhancement and marginal vascular signs. Histologically, the tumors showed two patterns: surface cuboidal cells and round stromal cells grouped in papillary, sclerotic, solid, and hemorrhagic patterns. The pattern of expression of TTF-1 and EMA was diffusely positive in both cell types, while CK7, SPA, and novel aspartic proteinase A (Napsin A) were mainly positive in surface cuboidal cells. Ki-67 expression remained low (1 to 5%). Lung adenocarcinoma was present in three patients. Follow-up was available for no recurrence/metastasis.

**Conclusion:**

PSP is a rare tumor of the lung with an indolent behavior, whose imaging and pathological characteristics might help in the differential diagnosis of other pulmonary nodules.

## 1 Introduction

Pulmonary sclerosing pneumocytoma (PSP) is a rare lung tumor that originates from type II alveolar epithelium (1. 11). It is mostly regarded as a benign or low-grade malignant lesion. It lacks specificity in clinical and imaging features and is easily confused with benign and malignant nodules, such as peripheral lung adenocarcinoma and hamartoma. The majority of the previous reports were single-case or small-sample retrospective studies. The systematic understanding of its imaging spectrum, histological composition, and immunophenotype is still insufficient, and the follow-up data are also limited (2). This study provides three new clinically relevant findings when compared with previously reported PSP series: (1) detailed correlation of CT imaging manifestations with predominant histologic patterns, (2) analysis of PSP cases occurring in the same lobe as lung adenocarcinoma, and (3) integrated follow-up imaging–pathology data on surgically treated patients in a contemporary single-center cohort. The results could help to improve the diagnosis of PSP in complex cases of pulmonary nodules.

## 2 Materials and methods

### 2.1 Research design and case sources

This study is a retrospective, single-center case series with a blinded imaging review performed separately. The subjects were 22 patients hospitalized at the First Affiliated Hospital of Bengbu Medical University from January 2022 to August 2025 who underwent surgical resection and were pathologically confirmed to have pulmonary sclerosing pneumocytoma (PSP). The inclusion criteria were as follows: ① postoperative pathology met the diagnostic criteria in the fifth edition of the WHO Classification of Thoracic Tumors and ② clinical records, imaging, and pathological data were complete. The exclusion criteria were as follows: ① primary solid malignant tumors at other sites and ② hematological malignancies. Among the 22 patients, there were 3 male patients and 19 female patients (male-to-female ratio ≈ 1:6.3). Age ranged from 25 to 77 years, with a mean age of 54.4 years. Relevant clinical and pathological information was extracted from the electronic medical record and pathology information systems. This study was approved by the Ethics Committee of the tertiary referral center [Lun Ke PI Zi (2024) No. 201] and complied with medical ethical standards. In 2024, prior to formal data collection and analysis, ethics approval was obtained. The patients included were those treated from January 2022 to August 2025, and the final follow-up/data-lock date was December 2025.

### 2.2 Contents of clinical data collection

General information was extracted from the electronic medical record system and follow-up forms, including sex, age, admitting department, comorbidities (hypertension, diabetes, coronary heart disease, and other comorbid conditions), smoking history, and history of lung disease. The history of smoking was also taken and divided into current smokers, former smokers, and never smokers. The mode of detection was recorded (pulmonary nodules/space-occupying lesions found on physical examination or presentation after symptom onset). Symptoms included dry cough, expectoration, chest/back discomfort, blood-streaked sputum, and chest tightness. Preoperative laboratory results were also collected, with serum tumor markers (carcinoembryonic antigen, cytokeratin 19 fragment, neuron-specific enolase, squamous cell carcinoma antigen, and gastrin-releasing peptide precursor) summarized for some patients to describe their abnormalities and associated clinical characteristics (3).

### 2.3 Imaging examinations and evaluation methods

All patients underwent chest CT examinations before the operation, including thin-slice (with a slice thickness of 1.0–1.5 mm) plain scan or plain scan combined with enhanced scan. The marginal vessel sign was delimited (4) as the vessels that enter the lesion margin or abut on the lesion margin compared to the contrast-enhanced CT. Some image data from other hospitals were uniformly imported into the PACS system of the tertiary referral center. The images were independently analyzed by two physicians with over 10 years of experience in chest imaging diagnosis, who were blinded to the pathological diagnosis, in a double-blinded fashion. If there was disagreement, consensus was reached through discussion. The content of the film reading includes the location, size, shape, boundary, density, and internal components (calcification, cavities, and other internal components) of the lesion, as well as adjacent structural changes, such as marginal vascular sign, halo sign, lobulation, small spicules, pleural traction, bronchial compression, and hilar or mediastinal lymph node enlargement. The various indicators are summarized in a CT imaging feature table for subsequent analysis (5).

### 2.4 Pathological and immunohistochemical detection methods

All surgically resected lung tissue specimens were fixed in 10% neutral formaldehyde, routinely dehydrated, and paraffin-embedded. Sections (4 μm) were prepared for hematoxylin–eosin (HE) staining and immunohistochemistry. Immunohistochemistry was performed using the two-step Elivision™ plus method. After deparaffinization and rehydration, sections underwent high-pressure antigen retrieval in an EDTA buffer at pH 8.0. Endogenous peroxidase was blocked with 3% hydrogen peroxide, followed by incubation with primary antibodies. The sections were then incubated with a universal secondary antibody, developed with DAB, counterstained with hematoxylin, dehydrated, and mounted. Antibodies included TTF-1, SPA, EMA, CK7, broad-spectrum CK, Napsin A, vimentin, SMA, S-100, and Ki-67. All primary antibodies (TTF-1, SPA, EMA, CK7, broad-spectrum CK, Napsin A, vimentin, SMA, S-100, Ki-67) and chromogenic kits were purchased from Fuzhou Maixin Biotechnology Development Co., Ltd. (Fuzhou, Fujian, China). Two senior pathologists, blinded to clinical data, independently evaluated the staining results. A semi-quantitative scoring system was used based on staining intensity and the proportion of positive cells, with a total score >2 considered positive (6). The results of immunohistochemical staining were assessed using a semi-quantitative scoring system. The intensity of staining was considered as follows: 0, negative staining; 1, weak staining; 2, moderate staining; and 3, strong staining. A proportion of positive tumor cells was graded as 0 in case of less than 5% positive cells, 1 in case of 5 to 25% positive cells, 2 in case of 26 to 50% positive cells, and 3 in case of over 50% positive cells. The proportion score and the staining intensity score were then added to give the final immunoreactivity score. A score of more than 2 was deemed to be a positive expression. Neuroendocrine markers, including neuron-specific enolase (NSE), synaptophysin (Syn), and chromogranin A (CgA), were additionally evaluated in six selected cases in which neuroendocrine differentiation was considered in the differential diagnosis based on morphologic features or intraoperative consultation findings.

### 2.5 Treatment methods and follow-up plans

All 22 patients underwent surgical resection. Three cases were complicated by lung adenocarcinoma, and postoperative pathology in the remaining cases confirmed PSP alone. All procedures were performed thoracoscopically. Surgical methods were selected according to lesion size, location, and relationship with surrounding structures, including wedge resection (*n* = 9), segmentectomy (*n* = 10), and lobectomy (*n* = 3). Among the three lobectomies, one was due to hypoplasia of the pulmonary fissure, one involved a lesion located at the junction of segment 3 of the right upper lobe, and one was performed because lung cancer was present in the same lobe. Lobectomy was chosen to ensure adequate margins and complete tumor resection.

The follow-up was conducted through outpatient reexamination combined with telephone follow-up, mainly recording the general condition of the patients, respiratory symptoms, and recurrence or distant metastasis on imaging. After discharge, all patients were regularly reexamined according to the conventional plan of the thoracic surgery department (7).

### 2.6 Statistical methods

Because of the retrospective design and limited number of cases, the analyses conducted in this study were descriptive and exploratory. Continuous variables (e.g., age, maximum lesion diameter, and CT attenuation values) were presented as mean ± standard deviation (SD) or median (interquartile range), as appropriate. Categorical variables (e.g., sex, symptom composition, lesion morphology, and immunohistochemical positivity) were summarized as the number of cases and percentage. Given the relatively small sample size, analyses were primarily descriptive; 95% confidence intervals (CIs) were provided for selected key proportions and means to clarify statistical uncertainty. Since this was primarily descriptive research, the statistical analyses were largely restricted to descriptive statistics, and no intergroup comparative analysis was conducted (8).

## 3 Results

### 3.1 CT imaging features

All 22 patients in this group completed chest CT examinations before the operation. The majority of lesions appeared as small pulmonary nodules or mass shadows in the lungs. Among them, 17 cases were round or irregular solid nodules, 3 cases were ground-glass nodules, 1 case was a part-solid nodule, and 1 case was a mass shadow. The diameters of the nodules ranged from 6 to 37 mm, with an average diameter of 14.43 mm.

Among the solid nodules, most of the lesions had clear boundaries. Eight cases showed lobulation or fine spicules, five cases had halo signs around them, and scattered calcification points were seen inside the nodules in three cases. Among the 17 cases of solid nodules, 16 cases showed the marginal vessel sign. The edges of the ground-glass nodules in three cases were slightly blurred, all accompanied by marginal vascular signs. Among them, two cases coexisted with lung adenocarcinoma lesions. One case was a partially solid nodule, and another was a mass shadow lesion with clear boundaries. The latter was located in the lower lobe of the right lung, with calcified points visible inside and multiple pulmonary bullae around it.

**Figure 1:**
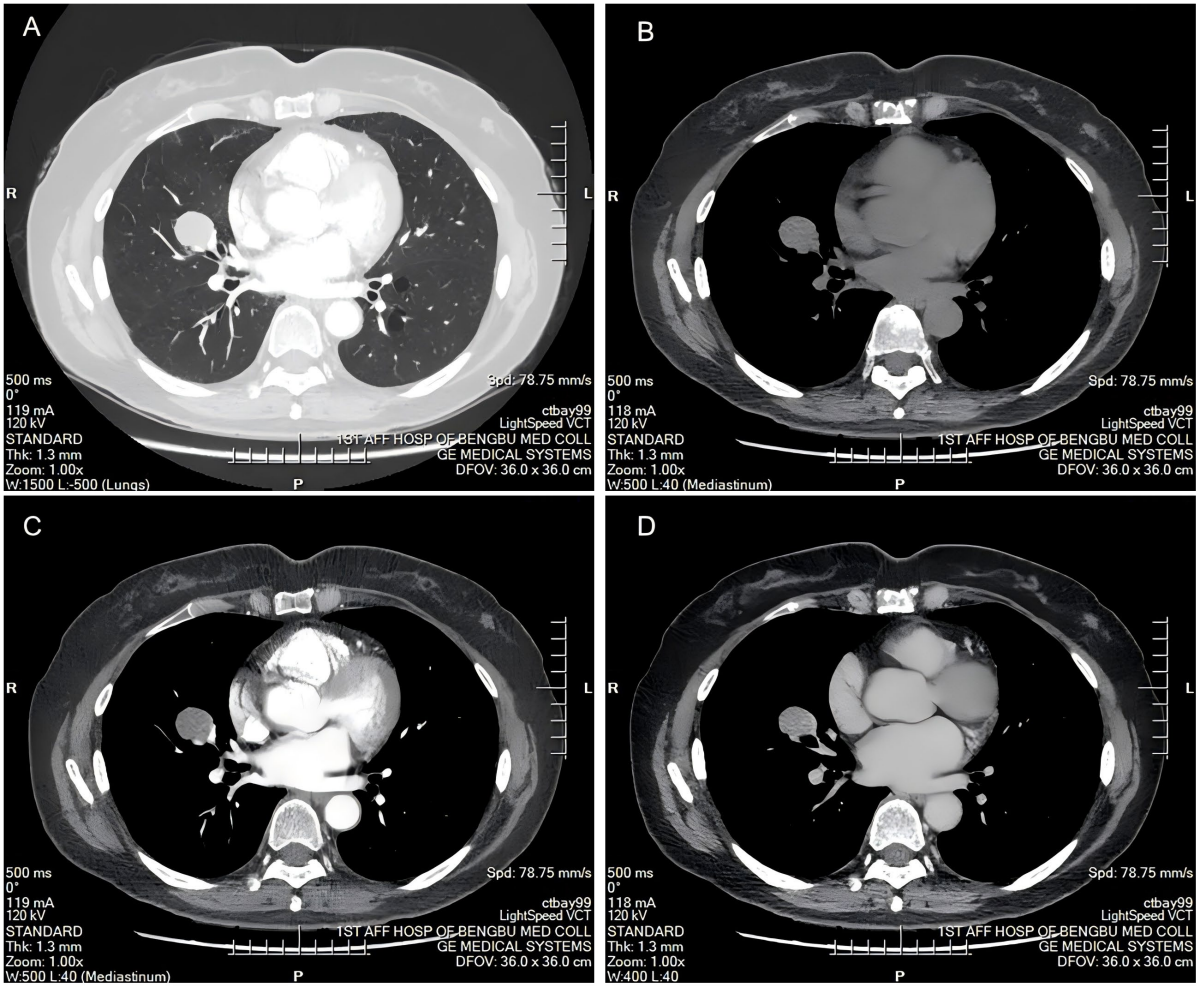
Typical solid nodule of pulmonary sclerosing pneumocytoma (PSP) on CT. White arrows indicate the lesion and marginal vessel sign. A well-circumscribed round solid nodule is visible in the medial segment of the right middle lobe. **(A)** Non-contrast lung window. **(B)** Non-contrast mediastinal window. **(C)** Arterial phase. **(D)** Venous phase showing progressive homogeneous enhancement.

**Figure 2:**
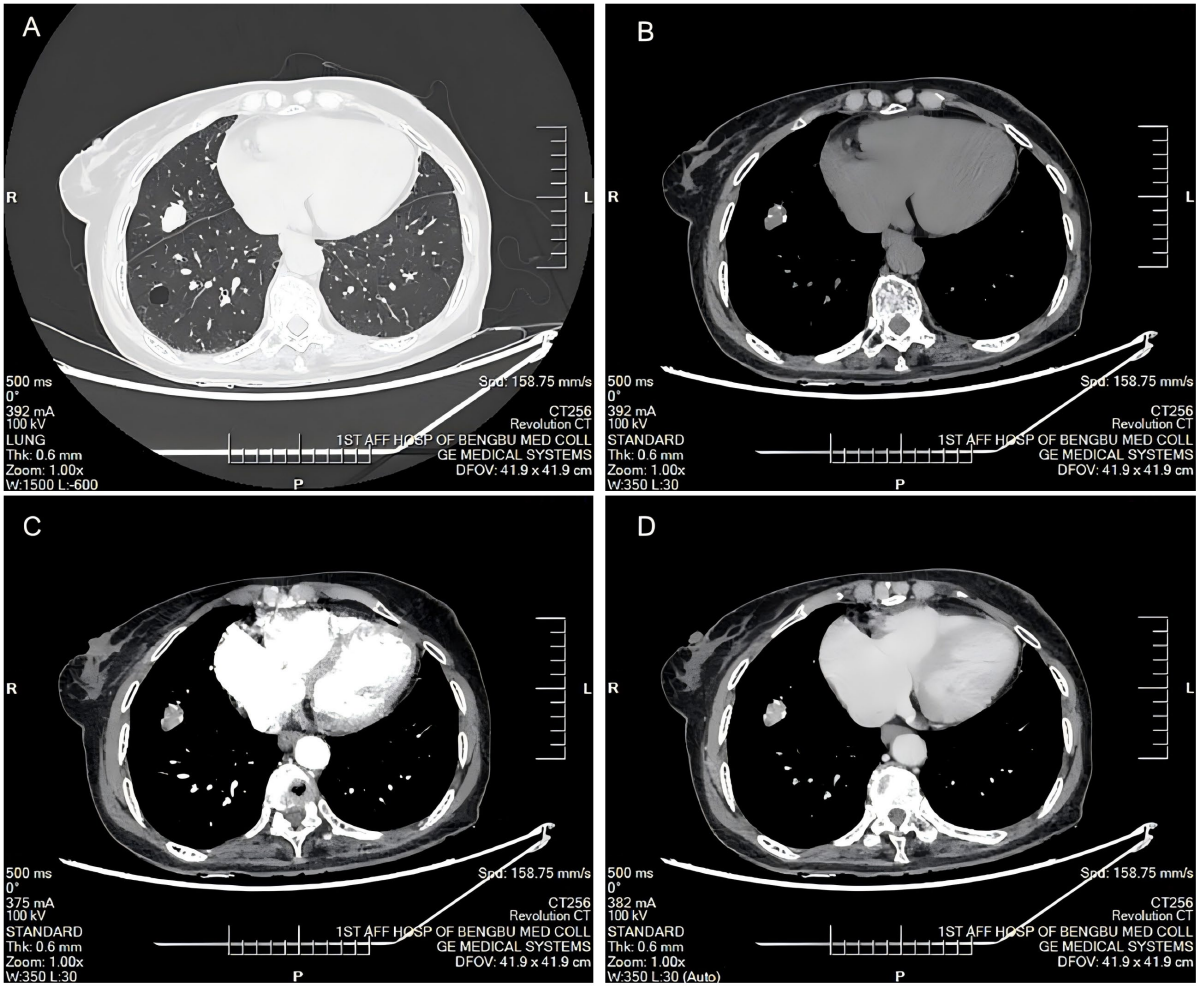
Irregular solid nodule of pulmonary sclerosing pneumocytoma (PSP) on CT. White arrows indicate the lesion, lobulation, and pleural indentation. An irregular solid nodule is seen in the lateral segment of the right middle lobe, with lobulation, fine spiculation, and punctate calcification. **(A)** Non-contrast lung window. **(B)** Non-contrast mediastinal window. **(C)** Arterial phase. **(D)** Venous phase showing mild-to-moderate progressive heterogeneous enhancement.

**Figure 3:**
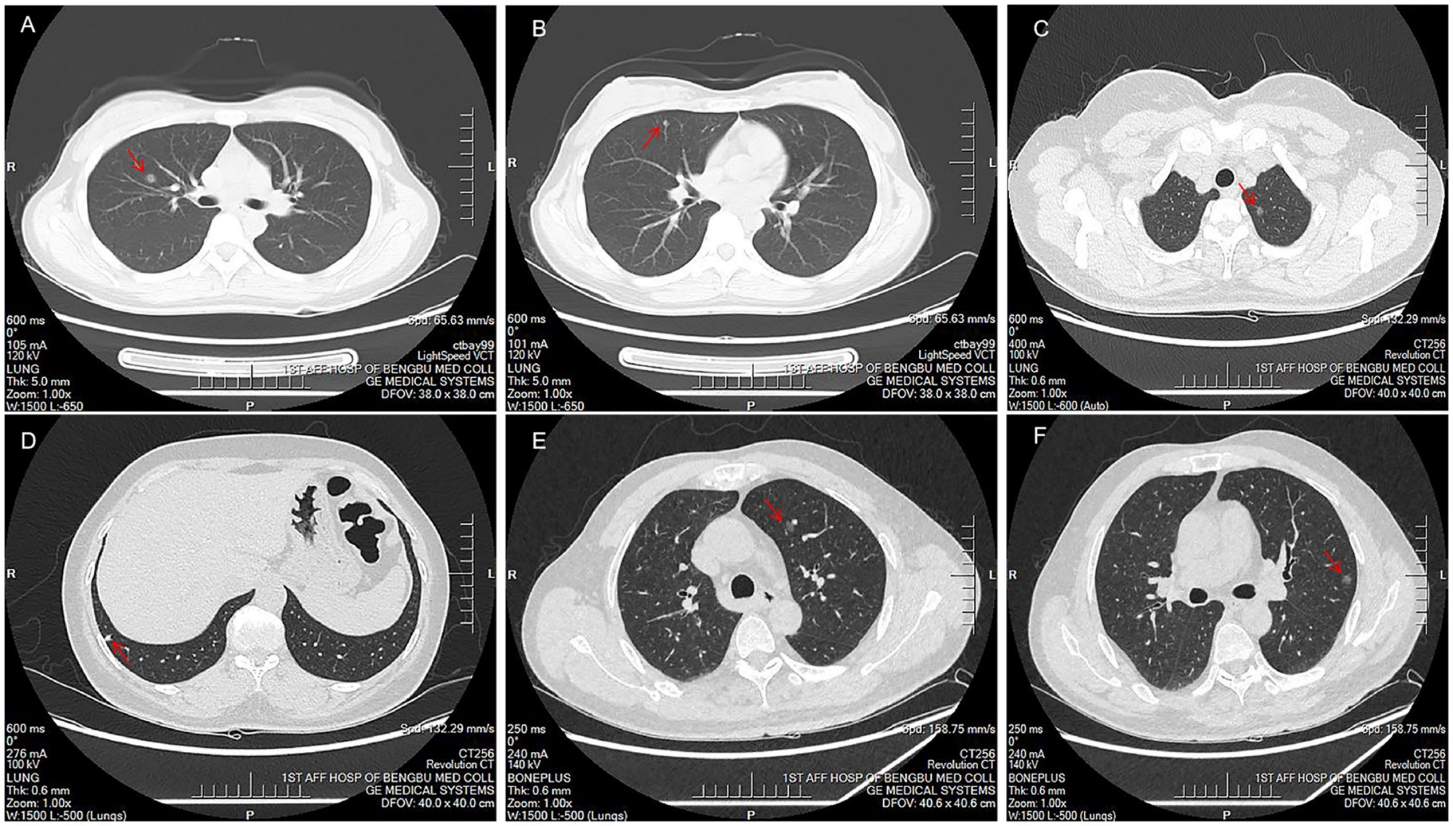
Ground-glass nodules of pulmonary sclerosing pneumocytoma (PSP) on CT. **(A, B)** A patient with PSP coexisting with lung adenocarcinoma: **(A)** A ground-glass nodule with blurred margins is seen in the anterior segment of the right upper lobe. **(B)** A ground-glass nodule is present in the middle lobe of the right lung. **(C, D)** Another patient: **(C)** A ground-glass nodule is observed in the apicoposterior segment of the left upper lobe. **(D)** A ground-glass nodule is present in the basal segment of the right lower lobe. **(E, F)** Another patient: Ground-glass nodules are observed in both the anterior and apicoposterior segments of the left upper lobe.

**Figure 4:**
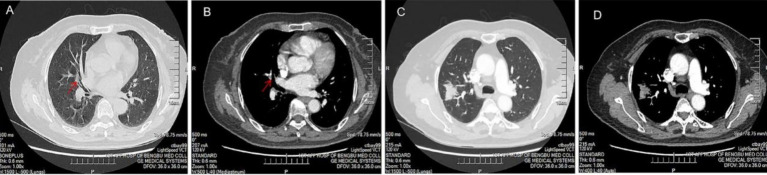
Coexisting pulmonary sclerosing pneumocytoma (PSP) and lung adenocarcinoma on CT. **(A-D)** One patient with a 7.6 mm × 6.1 mm nodule in the right middle lobe showing a marginal vessel sign; a separate upper-lobe mass was confirmed as lung adenocarcinoma.

Thirteen patients underwent plain scan plus enhanced CT examination. In 13 patients, contrast-enhanced CT was performed, of whom complete arterial and venous phase attenuation measurements were available for 11 patients. In the other two cases, the external information included in the imaging database was incomplete, and standardized quantitative attenuation analysis was not possible. The average CT value of the plain scan was 26.9 HU, and the average CT values in the arterial phase and venous phase were 56.8 HU and 75.9 HU, respectively. The majority of lesions demonstrated progressive enhancement on contrast-enhanced CT. Among the 11 cases, 8 cases showed uniform enhancement and 3 cases showed non-uniform enhancement. The detailed CT imaging features of the 22 patients are shown in Table 1. The typical CT manifestations of different types of lesions are shown in Figures 1–4.

**Table 1:** Summary of demographic and CT characteristics of pulmonary sclerosing pneumocytoma (PSP) (*n* = 22).

Item	Value
Sex (female), *n*/*N* (%; 95% CI)	19/22 (86.4%; 66.7–95.3)
Age (years), mean ± SD (95% CI)	54.4 ± 14.6 (47.9–60.8)
Lesion location, *n* (%)	Right upper lobe 3 (13.6%); right middle lobe 4 (18.2%); right lower lobe 7 (31.8%); left upper lobe 4 (18.2%); left lower lobe 4 (18.2%)
Lesion diameter (mm)* mean ± SD (95% CI)	14.4 ± 6.2 (11.7–17.2)
Lesion margin (clear), *n*/*N* (%; 95% CI)	18/22 (81.8%; 61.5–92.7)
Enhanced CT attenuation (*n* = 11) † non-contrast (HU), mean ± SD (95% CI)	26.9 ± 8.1 (21.5–32.3)
Enhanced CT attenuation (*n* = 11) † arterial phase (HU), mean ± SD (95% CI)	56.8 ± 11.2 (49.3–64.4)
Enhanced CT attenuation (*n* = 11) † venous phase (HU), mean ± SD (95% CI)	76.0 ± 13.4 (67.0–85.0)

*Lesion diameter was calculated as (long axis + short axis)/2 from CT measurements (consistent with “14.43 ± 6.20 mm”). †Enhanced CT attenuation values were available in 11 cases with complete arterial/venous measurements.

### 3.2 Pathological morphological characteristics

Generally, tumors are mostly located within the lung parenchyma, are mostly round or nearly round in shape, with a few having irregular shapes. They have a clear boundary from the surrounding lung tissue, and in some cases, a relatively complete fibrous capsule can be seen. The cross-section shows diverse colors, which can be grayish white, yellowish brown, or dark red. The color differences are related to the cellular components within the tumor, lipid content, and the degree of old hemorrhage. The texture varies from slightly soft to medium hardness. Scattered calcification foci can be seen in some lesions, and generally, no obvious necrosis is observed.

**Figure 5:**
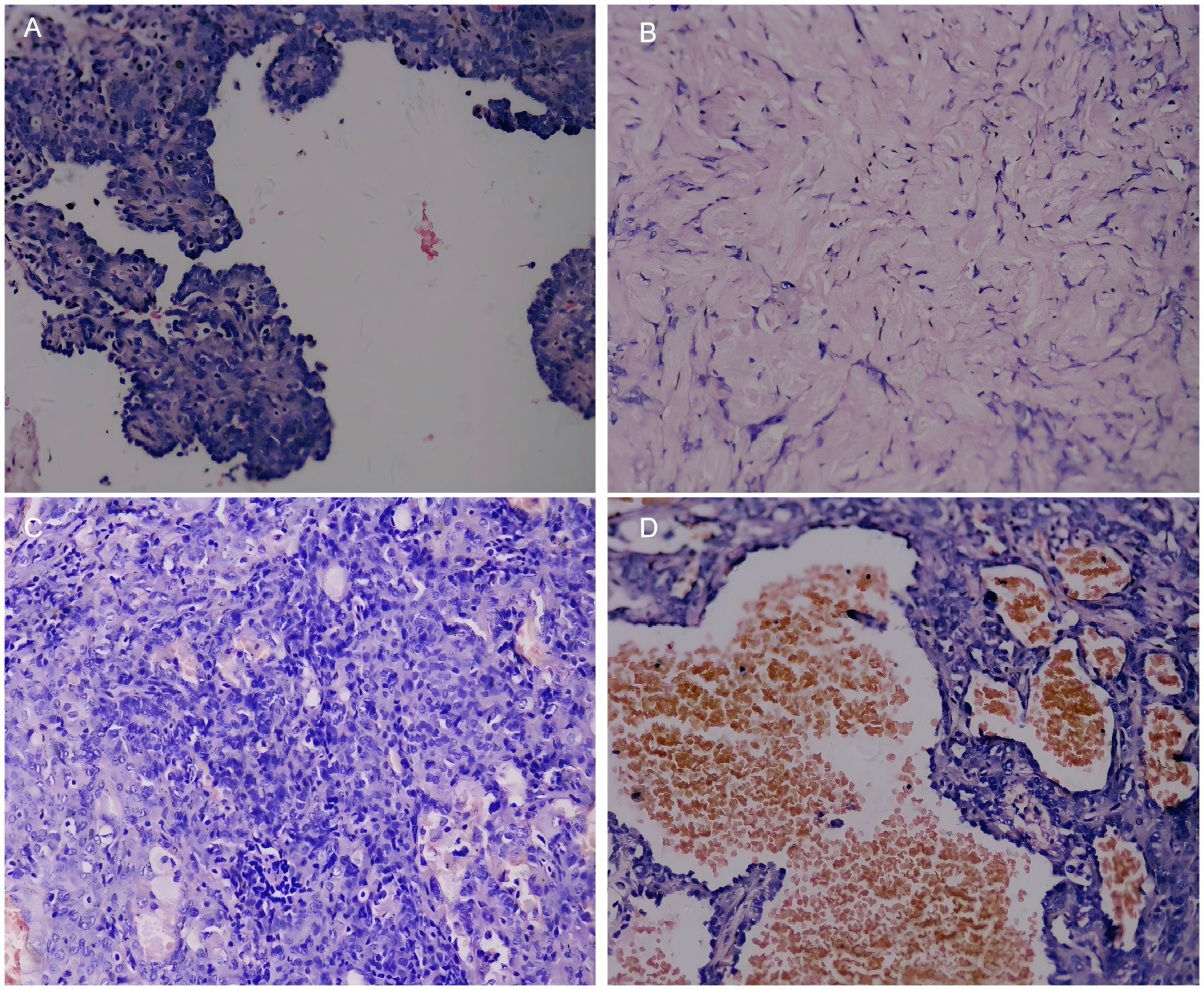
Representative histologic morphology of pulmonary sclerosing pneumocytoma (PSP) (HE staining). **(A)** Papillary pattern, **(B)** sclerotic pattern, **(C)** solid pattern, and **(D)** hemorrhagic pattern. Embedded scale bars = 100 μm.

Under the microscope, typical “biphasic” cell composition can be seen, mainly consisting of surface cuboidal cells lining the papillary or lacunar surface and round (or polygonal) interstitial cells located within the interstitium. Two types of cells form four structural patterns in different proportions: papillary, sclerosing, solid, and hemorrhagic. The papillary region is mostly located around the tumor, and the surface cells are neatly arranged. The sclerotic area is more common at the center of the lesion, with hyaline degeneration of the fibrous interstitium being dominant. The solid area is composed of circular interstitial cells distributed in patches. Hemangiomatous cavities filled with red blood cells are commonly seen in the bleeding area. The tumor cells have mild atypia, and mitotic figures are extremely rare. No definite necrotic foci have been observed. The overall morphology suggests a benign or low-grade malignant biological behavior. The typical histological morphology is shown in Figure 5.

### 3.3 Immunohistochemical expression profile

Immunohistochemical detection was performed on all 22 tumor specimens. Both TTF-1 and EMA showed diffuse strong positivity in surface cuboidal cells and round stromal cells, supporting that the tumor originated from alveolar epithelioid cells. SPA was positive in all surface cuboid cells but negative in round interstitial cells, while vimentin was positive in all round interstitial cells. Only four cases showed weak positivity for surface cuboid cells, suggesting differences in the differentiation lineage between the two types of cells.

In terms of keratin-related markers, CK7 and broad-spectrum CK are positive in most surface cuboidal cells, and in some cases, they can also be expressed in round stromal cells. Napsin A and SPA were mainly confined to surface cuboidal cells, with only a few round stromal cells being weakly positive, suggesting that they retained the differentiation characteristics of type II alveolar epithelium. In six selected cases, neuroendocrine markers (NSE, Syn, and CgA) were examined. All were negative for Syn and CgA; two were focal weak positive for NSE. There was no definitive immunophenotype of neuroendocrine cells. The Ki-67 proliferation index was generally low. Among the two types of cells, the majority were positive at 1 to 5%, which was consistent with the result that no recurrence or metastasis was observed during the follow-up, supporting its low-grade malignant biological behavior.

**Figure 6:**
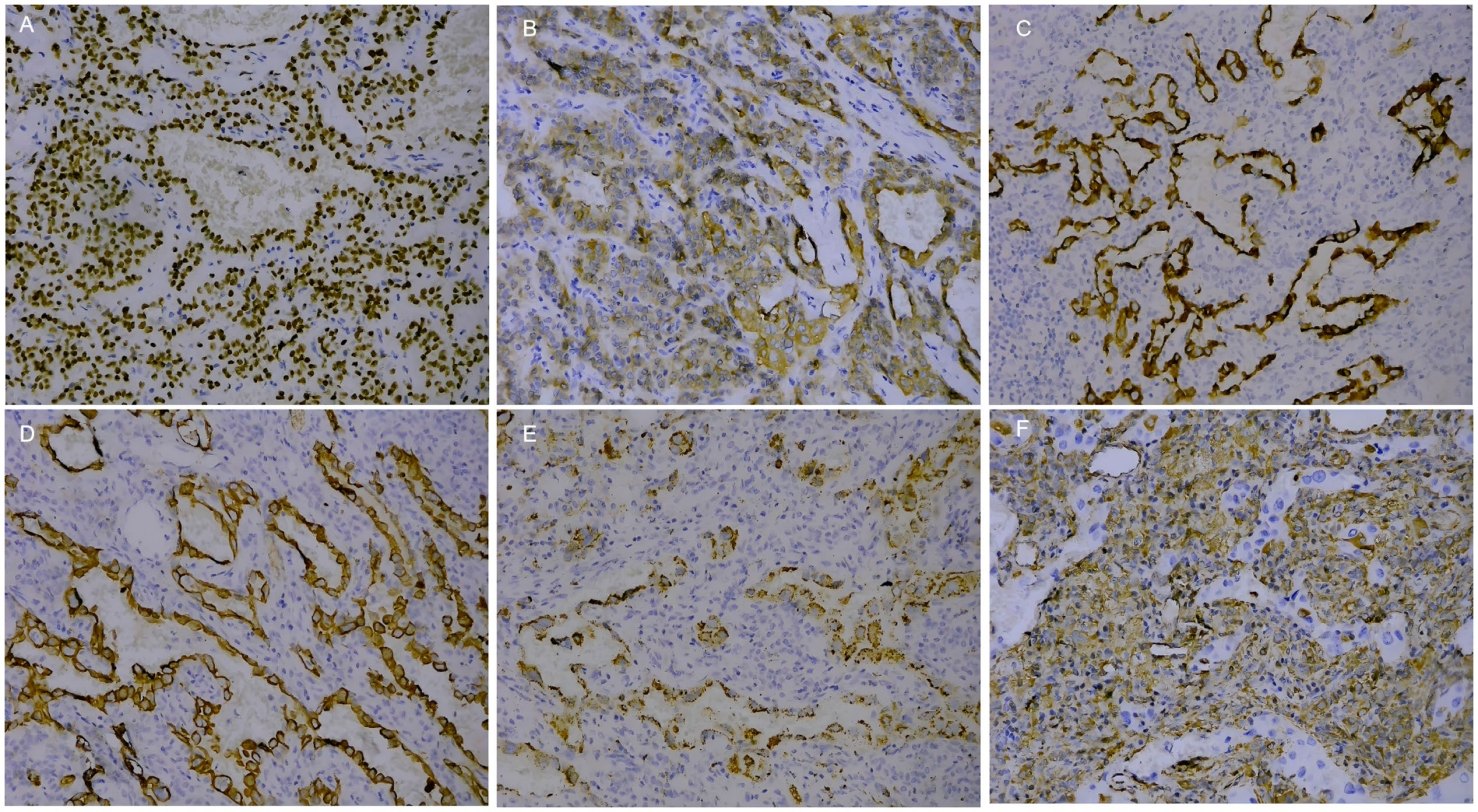
Representative immunohistochemical staining patterns of pulmonary sclerosing pneumocytoma (PSP). Scale bar = 100 μm **(**applies to all panels **A–F)**.

The specific positive rates of each immune marker are shown in Table 2, and the typical immunohistochemical staining patterns are presented in Figure 6.

**Table 2:** Immunohistochemical results of 22 cases of pulmonary sclerosing pneumocytoma (PSP).

Immune markers	Positive cuboidal cells on the surface	Round interstitial cells are positive
TTF-1	22	22
SPA	22	0
EMA	22	22
CK7	19	0
CK	19	5
Napsin A	17	0
Vimentin	4	22
SMA	0	0
S-100	0	0
Ki-67 (1%+)	13	14
Ki-67 (2%+)	5	4
Ki-67 (3%+)	2	2
Ki-67 (5%+)	2	2

Neuroendocrine markers (NSE, Syn, and CgA) were evaluated only in six selected cases for differential diagnostic purposes. TTF-1, thyroid transcription factor-1; SPA, surfactant protein A; EMA, epithelial membrane antigen; CK, cytokeratin; Napsin-A, novel aspartic proteinase A; SMA, smooth muscle actin; S-100, S100 calcium-binding protein; Ki-67, proliferation marker Ki-67.

### 3.4 Exploratory correlation between CT features and histologic patterns

Papillary and hemangiomatous patterns were the most frequent histologic patterns of PSP lesions, characterized by progressive lesion enhancement on CT, suggesting relatively rich vascular stroma. Microlesions with sclerotic areas or hemorrhages were more likely to be found in lesions with “non-uniform” enhancement or “intracalcification.” Ground-glass nodules were correlated with an equivalent relatively limited solid architectural component and mild stromal sclerosis.

### 3.5 Surgical methods and perioperative outcomes

All 22 patients with pulmonary sclerosing pneumocytoma (PSP) in this group received surgical resection treatment, among whom three cases were simultaneously complicated with lung adenocarcinoma lesions. All cases underwent surgical operations under thoracoscopy. Wedge resection, segmental resection, or lobectomy was selected based on the size, location of the lesion, and its relationship with blood vessels and bronchi. During the operation, rapid frozen pathological assessment of the lesion and necessary lymph node sampling were routinely performed. The majority of solitary and peripheral small nodules could be treated with wedge-shaped or segmental resection to obtain sufficient margins. For lesions located near the interlobar fissures or combined with lung cancer in the same lobe, lobectomy is performed to achieve radical tumor treatment. The overall postoperative recovery was good, and the average postoperative hospital stay was 4.1 days. Intraoperative frozen section examination helped guide surgical decision-making by confirming benign pulmonary lesions consistent with PSP.

### 3.6 Follow-up, prognosis, and analysis of special cases

All 22 patients completed the follow-up assessment through outpatient reexamination or telephone follow-up after the operation. The follow-up period was calculated from the date of the operation. By the end of the follow-up, six cases were followed up for 3 years, eight cases for 2 years, four cases for 1 year, and the remaining four cases for 6 to 11 months. The overall median follow-up time was approximately 24 months. The mean follow-up duration was 24.0 ± 9.6 months. During the follow-up period of all cases, no local recurrence or distant metastasis was observed, nor were there any tumor-related deaths. Among the three patients with lung adenocarcinoma, two had ground-glass nodular adenocarcinoma lesions in the same lobe, and one had a small nodular adenocarcinoma in the same lung lobe. All were completely resected during the operation with negative resection margins. No recurrence of or progression of adenocarcinoma was observed during the 1 to 3 years of follow-up. However, considering that the follow-up time for some patients is still short, it is necessary to extend the observation in the future to further verify their long-term prognosis.

This part of the cases suggests that PSPs themselves mostly grow indolently. However, in the context of multiple nodules or ground-glass lesions, the possibility of coexisting lung adenocarcinoma still needs to be considered. Preoperative assessment and intraoperative pathology require thorough sampling and differentiation of suspected malignant lesions (9). Given the relatively short follow-up times in several patients (<12 months), the long-term recurrence risk was not sufficiently evaluated.

## 4 Discussion

### 4.1 Clinical overview

PSP is a rare indolent lung tumor that originates from type II alveolar epithelium. Epidemiological data show that middle-aged women and non-smokers are the main affected groups. The epidemiological patterns of PSP, in which these demographic features are observed, mostly affect middle-aged women (15, 19). The sex distribution of our cohort (86.4% female patients) and the mean patient age of 54.4 years are similar to those of larger retrospective studies, such as Chen et al. (3), which also reported a similar sex distribution and age range. These results also confirm the hypothesis that hormonal or genetic influences can be the causes of PSP development, but the mechanism itself is still not clear. There were 15 patients over 50 years old (68.18%), which was basically consistent with the age and gender distribution reported in the previous case series. Among them, 16 cases had pulmonary nodules incidentally discovered during routine physical examinations or chest CT scans, while the remaining patients mostly presented with non-specific symptoms such as mild cough, chest tightness, or discomfort in the chest and back. The preoperative serum tumor marker tests were mostly normal, and only two cases showed elevated NSE, suggesting that the clinical and laboratory characteristics of this disease were atypical and were typically identified incidentally during imaging screening (10).

### 4.2 Pathology and immunity

The specimens in this group are mostly well-defined, round, or nearly round nodules. Under the microscope, two types of components, namely, surface cuboidal cells and round interstitial cells, can be seen, which form papillary, sclerotic, solid, and hemorrhagic structures in different proportions. The majority of lesions consist of multiple components. The cell atypia is mild, mitotic figures are rare, and no definite necrotic foci are observed. Morphologically, these lesions exhibit the characteristics of indolent tumors. In terms of immunophenotype, TTF-1 and EMA were diffusely positive in both types of cells. CK7 and Napsin A were mainly expressed in the surface cuboidal cells. Vimentin was mainly positive in round stromal cells. SMA and S-100 were both negative. It forms a relatively typical combination of “TTF-1/EMA double positive and clearly differentiated epithelial/mesenchymal markers,” which is helpful for differentiating from lung adenocarcinoma and other benign nodular lesions. The Ki-67 index of this group was mostly between 1 and 5%, which was consistent with the follow-up results of no recurrence or metastasis, providing support for its low proliferation and low-grade malignant biological behavior.

The biphasic cell structure of PSP reflects the differentiation of primitive respiratory epithelial cells (12, 14). The positivity of TTF-1 and EMA in both surface and stromal cells suggests a common cellular origin in epithelial cells, whereas the SPA, CK7, and vimentin divergence suggests cellular differentiation pathways. The low Ki-67 proliferation index, which was consistently low in this study, supports the indolent biological behavior and low recurrence risk of PSP.

### 4.3 Imaging manifestations

The marginal vessel sign is one of the most typical imaging features of PSP. Histologically, this characteristic may reflect an increase in peritumoral capillary networks and enhanced vascularity within the tumor stroma. The gradual increase in contrast enhancement observed on CT imaging is probably attributable to the accumulation of contrast material in the sclerotic and vascular components of the lesion. The identification of this enhancement pattern can help radiologists differentiate PSP from primary lung adenocarcinoma (13), which exhibits more common heterogeneous enhancement and invasive morphology.

This group of PSPs mostly presented as small nodules in the lungs, including round or irregular solid nodules, a small number of ground-glass nodules, and part-solid nodules. The nodules generally have clear boundaries, and the marginal vascular sign is relatively common. Some are accompanied by lobulation, small spicules, and halo signs. Contrast-enhanced CT mostly shows progressive enhancement. These findings are basically consistent with the imaging characteristics of “clear boundaries, small nodules, marginal vascular signs, and progressive enhancement” reported in previous case series.

It is worth noting that in this group, there was a case in which ground-glass nodules were combined with adenocarcinoma in the same lung lobe, which makes the characterization of multiple ground-glass or small nodule lesions on imaging more complicated and can be easily mistaken for multiple primary lung adenocarcinomas or adenocarcinoma with satellite lesions. Combined with the characteristics of the mostly indolent growth of the lesion, prominent marginal vascular signs, and the lack of typical pleural depression and lymph node enlargement seen in invasive adenocarcinoma, it is helpful to enhance the vigilance for PSP before surgery. However, it is still difficult to completely distinguish it from some peripheral lung adenocarcinomas on imaging, and further diagnosis relies on pathology and immunohistochemistry.

### 4.4 Diagnosis and differentiation

PSPs remain difficult to accurately identify before surgery. Although most cases in this cohort presented as well-defined small nodules with typical marginal vascular signs and progressive enhancement, and postoperative pathology revealed biphasic cells with characteristic immunophenotypes, preoperative distinction from peripheral lung adenocarcinoma and other lesions based solely on imaging and routine examinations remains challenging. Therefore, postoperative pathology and immunohistochemistry were often relied on for diagnosis, which was consistent with the conclusion in the literature that “the preoperative misdiagnosis rate was relatively high.”

The key points in the differential diagnosis lie in distinguishing PSP from peripheral lung adenocarcinoma, hamartoma, and tuberculous nodules. Peripheral lung adenocarcinoma typically progresses more rapidly and is more prone to invasive manifestations such as obvious spiculation, pleural depression, and the bronchial inflation sign. Although TTF-1, CK7, and Napsin A can also be positive, it lacks the biphasic pattern of “double positive TTF-1/EMA and strong positive vimentin in interstitial cells.” Hamartomas are commonly characterized by fatty components and “popcorn” calcification. Tuberculous nodules are often accompanied by cavities, satellite foci, and systemic toxic symptoms. A comprehensive assessment of the clinical background, imaging features, and biphasic immunophenotype can help reduce the misdiagnosis rate.

The distinction between PSP and peripheral lung adenocarcinoma is a significant clinical problem (18, 20). Even though both lesions can present TTF-1 positivity, PSP is usually characterized by a biphasic cellular structure and a low Ki-67 index, and adenocarcinoma is characterized by greater proliferation and invasiveness. A combination of imaging findings, especially clear borders, marginal vessel signs, and gradual enhancement, along with immunohistochemical analysis of markers (TTF-1, Napsin A, SPA, and vimentin), can help to improve the accuracy of preoperative diagnosis and minimize excessive surgical resection.

### 4.5 Treatment and outcomes

It is currently widely believed that surgical resection is the main treatment for PSP. Both the literature and the data of this group show that the majority of patients can achieve good control through thoracoscopic wedge resection or lobectomy. Only when the lesion location is complex, the pulmonary fissure is poorly developed, or there is a combination of lung adenocarcinoma, is a total lobectomy required to ensure the safety of the resection margin and to ensure radical cure of malignant lesions.

The average postoperative hospital stay of the 22 patients in this group was 4.1 days. The longest follow-up was 3 years. No local recurrence or distant metastasis was observed, and there was no tumor-related death. Patients with lung adenocarcinoma also achieved good control after simultaneous resection of the suspected lesion. Based on previous literature and the results of this study, PSP as a whole exhibits a low-grade malignant biological behavior and slow growth (16, 17). In clinical practice, for peripheral small nodules with imaging and pathological features that match this disease, under the premise of fully assessing the risk of coexisting lung adenocarcinoma and ensuring the safety of the resection margin, it is more reasonable to give priority to limited resection methods, such as wedge or segmental resection, that preserve lung parenchyma. Only when the lesion is complex or combined with malignant tumors does the resection range need to be expanded. It can avoid over-treatment while taking into account the long-term prognosis.

The presence of both the PSP and lung adenocarcinoma in three patients highlights the importance of carefully evaluating multiple pulmonary nodules. The lesions can be collision tumors and not malignant transformation. Consequently, intraoperative pathological examination and long-term follow-up should be conducted thoroughly when nodules of varying imaging features are involved. Given the indolent biological behavior of PSP, some small asymptomatic lesions identified on screening may be managed conservatively with close follow-up. However, surgical resection remains necessary when malignancy cannot be definitively excluded.

Preoperatively, the presence of both patterns is important to consider, as they can both appear as ground-glass or as small peripheral nodules on a CT scan. For this group, pre-operative diagnosis of independent synchronous lesions versus invasive adenocarcinoma was challenging. Therefore, intraoperative frozen section analysis was very important in defining the extent of resection (21). A limited resection may be sufficient if a lesion is isolated, but if an adenocarcinoma is suspected, a lobectomy should be performed to ensure oncologic safety and sufficient margins. The biological connection between PSP and coexisting adenocarcinoma is unknown at this time and is subject to further multicenter studies.

### 4.6 Study limitations

This research is limited in several ways. To start with, the relatively small sample size and the retrospective, single-center design of the study can be subject to selection bias. Second, the study lacks a comparative control group, which makes it difficult to conduct inferential statistical analyses. Third, it has not been followed up extensively, since some of the patients have short follow-up periods. More multicenter studies that involve larger cohorts with longer follow-up durations are necessary to confirm these findings. Because there was no control cohort representing lung adenocarcinoma or benign pulmonary nodules or ground-glass opacity-dominant lesions, the ability to conduct direct comparisons was limited, and diagnostic specificity was not fully assessed. The limitations might decrease the generalizability of the results and could also increase the likelihood of selection bias due to the fact that only cases that had been surgically treated and pathologically confirmed were included.

## 5 Conclusion

Pulmonary sclerosing pneumocytoma (PSP) is a rare tumor of the lung, primarily seen in middle-aged women, and may have clinical manifestations as a slowly growing, well-circumscribed pulmonary nodule seen on computed tomography (CT). A biphasic histological pattern and immunohistochemistry may help differentiate PSP from other pulmonary lesions. The results of this retrospective, single-center study without a comparative control group and with relatively short follow-up in some of the patients, however, should be interpreted with caution when drawing diagnostic and prognostic conclusions. There is a need for larger, multicenter studies with longer follow-up and comparative cohorts to further validate these observations.

## Data Availability

The original contributions presented in the study are included in the article/supplementary material, further inquiries can be directed to the corresponding author.
